# Antibiotic use and adherence to Finnish treatment guidelines in pediatric acute otitis media

**DOI:** 10.1007/s00431-026-06964-w

**Published:** 2026-04-27

**Authors:** Lisa Kulppi, Ville Peltola, Paula A. Tähtinen

**Affiliations:** 1 Department of Paediatrics, Vaasa Central Hospital, Wellbeing Services County of Ostrobothnia, Hietalahdenkatu 2-4, 65130 Vaasa, Finland; 2 Department of Paediatrics and Adolescent Medicine, Turku University Hospital and University of Turku, Savitehtaankatu 5, 20520 Turku, Finland

**Keywords:** Acute otitis media, Treatment, Guideline adherence, Children

## Abstract

Finnish guidelines recommend antimicrobial treatment for acute otitis media (AOM), with both amoxicillin and amoxicillin-clavulanate as equally acceptable first-line treatment options. This study aimed to investigate in what proportion different antimicrobial agents are prescribed for children with AOM. Our hypothesis was that amoxicillin is the most frequently prescribed antimicrobial agent. This register-based cohort study included all children less than 16 years of age with a diagnosis of AOM in two Finnish hospitals in 2023. Data on children’s demographics, AOM symptoms, findings and treatment were collected individually from the patient charts. The primary outcome was the proportion of children who were prescribed amoxicillin, amoxicillin-clavulanate, or other antimicrobial agents for the treatment of AOM. The antimicrobial treatment choices and durations were compared between Turku and Vaasa and reported as percentage differences with 95% confidence intervals. A total of 1,240 children in Turku and 393 children in Vaasa were included in this study. Among these, 80.9% (1,003/1,240) and 80.7% (317/393) were prescribed amoxicillin for the treatment of AOM in Turku and Vaasa, respectively. Amoxicillin-clavulanate was the second most used antimicrobial treatment, prescribed to 10.1% (125/1,240) and 8.7% (34/393) of the children in Turku and Vaasa, respectively. Most of the children with AOM were prescribed a short 5-day course of treatment.

*Conclusion*: A short 5-day course of amoxicillin was the most frequently prescribed antimicrobial treatment for children with AOM. The adherence to AOM guidelines appears to be high in the two hospitals studied.

**Table:** 

**What is Known:**
• *Amoxicillin and amoxicillin-clavulanate are recommended as first-line treatments for acute otitis media in Finland.* • *National data on real-world antibiotic choices have been limited.*
**What is New:**
• *Amoxicillin was the most frequently prescribed antibiotic in the two Finnish hospitals studied.* • *The most common treatment duration was a short 5-day course of treatment.*

• *Amoxicillin and amoxicillin-clavulanate are recommended as first-line treatments for acute otitis media in Finland.*

• *National data on real-world antibiotic choices have been limited.*

• *Amoxicillin was the most frequently prescribed antibiotic in the two Finnish hospitals studied.*

• *The most common treatment duration was a short 5-day course of treatment.*

## Introduction

Acute otitis media (AOM) is a common infection in childhood and the most frequent cause of antibiotic use among children [1]. Amoxicillin is considered effective, with a favorable safety profile and narrow-spectrum activity. It is widely recommended as the first-line antibiotic for AOM in most published guidelines, including many European countries, the United States (US) and Canada, although the recommended dose may vary [2–5]. In 1999, the Finnish Current Care Guidelines for the diagnosis and treatment of AOM initially recommended amoxicillin as the first-line treatment [6]. In the third revision, published in 2017, amoxicillin-clavulanate was added as an alternative first-line option. The recommended treatment is amoxicillin 40 mg/kg/day or amoxicillin-clavulanate 40/5.7 mg/kg/day divided into 2 or 3 doses for 5 to 7 days to children of all ages. For children with penicillin allergy, suitable alternatives include sulfamethoxazole-trimethoprim, azithromycin and clarithromycin. In cases where oral administration is not possible, for example due to vomiting, a single dose of ceftriaxone intramuscularly is an alternative treatment option. According to Finnish guidelines, antibiotic treatment is generally recommended when an AOM diagnosis is confirmed. Guidelines also provide the option for watchful waiting with a reassessment if the child is not clearly improving within 2–3 days. [7] Although Finland has these national guidelines, it remains unclear how AOM is treated in real life, in what proportion different antibiotics are used in the treatment of AOM and how consistently the Finnish guidelines are followed.

Our aim was to investigate the choice and duration of antibiotics prescribed for AOM in a cohort of children with AOM in two hospitals. Our hypothesis was that amoxicillin is the most frequently prescribed antimicrobial agent. We also compared the microbial prescribing patterns between the two hospitals to identify potential differences at the local level.

## Methods

This was a retrospective register-based study conducted at Turku University Hospital in Turku, Finland, and Vaasa Central Hospital in Vaasa, Finland. The study population consisted of children aged 0–15.99 years who were diagnosed with AOM between January 1, 2023, and December 31, 2023. We conducted a register search based on specific International Classification of Diseases 10th Revision (ICD-10) codes. We included children who had visited the Emergency Department (ED), for primary care or specialized medical care, or pediatric wards of Turku University Hospital or Vaasa Central hospital and had any of the following diagnostic codes: H66.0 (Acute suppurative otitis media), H66.4 (Suppurative otitis media, unspecified) and H66.9 (Otitis media, unspecified). We excluded children whose AOM diagnosis had been made prior to the hospital visit, who had already been included (duplicated visit entries) and children with an incorrect diagnosis code, i.e. the documented diagnosis code did not align with the diagnosis described in the medical record. We also excluded children with tympanostomy tubes or a concomitant diagnosis, i.e. bacterial co-diagnosis that affected the antimicrobial choice. Two separate registry searches were conducted in Turku and Vaasa, respectively, to identify eligible cases. An individual review of electronic patient charts was performed to acquire relevant data.

The primary outcome of this study was the proportion of children who were prescribed amoxicillin, amoxicillin-clavulanate, or other antimicrobial agents for the treatment of AOM. The secondary outcomes were the duration of the antimicrobial treatment, the frequency of watchful waiting and safety-net antibiotic prescriptions, visits within one week after the index AOM visit date and treatment failures, and in Vaasa Central Hospital, the specialty of the physician (data not available for the children in Turku). The duration was determined as total days of therapy. A safety-net antibiotic prescription meant that the guardian was given instructions to fill the antibiotic prescription only if the child’s symptoms persisted or worsened. Treatment failure was defined as a change of antimicrobial treatment within one week. The physicians were classified as general practitioners (GP), residents in Pediatrics, specialists in Pediatrics and ear, nose and throat (ENT) physicians, either a specialist or a resident in ENT. Background information was also collected on the patients’ sex, age, hometown, chronic diseases, previous AOM, symptoms, otoscopy findings, otorrhea (either reported or confirmed by examination), perforation of the tympanic membrane, the laterality of the infection, reported antimicrobial allergies and potential inpatient care. After the analyses for both hospitals were conducted separately, the antimicrobial results from Turku and Vaasa were compered.

### Statistics

The data were coded and handled anonymously, ensuring that individual patients could not be identified from the study. Patients’ personal information was only used when investigating details related to the diagnosis and treatment of their AOM from electronic medical records. The analyses for Turku and Vaasa were conducted in two separate environments within Auria’s Atolli, an operating environment designed for processing social and health care data requiring a high level of data security. The Finnish Social and Health Data Permit Authority verified the anonymization of the analysis. Categorical variables were summarized using frequencies and relative proportions (%), while continuous variables were described using means with standard deviations (SD) and medians with interquartile range (IQR). Data were analyzed using SPSS (IBM SPSS Statistics for Linux, version 27). Comparisons between Turku and Vaasa were conducted by calculating the percentage differences with 95% confidence intervals (CIs) with Microsoft Excel software.

### Ethics

The study protocol for this register-based study was approved by the Wellbeing Services County of Southwest Finland and the Wellbeing Services County for Ostrobothnia. According to Finnish legislation, approval from the Ethics Committee was not required, nor was individual informed consent of patients or their guardians necessary. The study was reported in accordance with the STROBE guidelines.

## Results

**Fig. 1:**
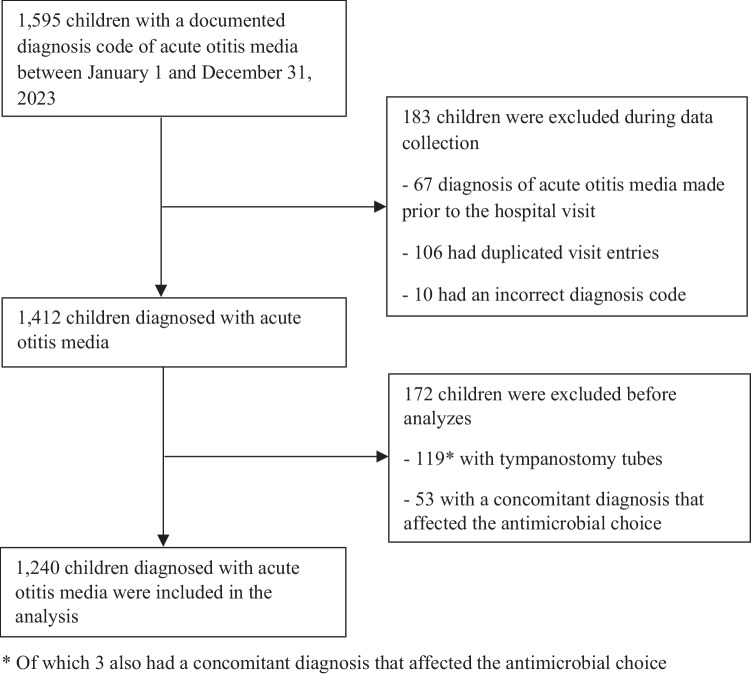
Figure 1 shows the flowchart of the study population in Turku. Of the 1,595 identified children with a diagnosis of acute otitis media in 2023, 183 children were excluded because the diagnosis had been made prior to the hospital visit, they had duplicated visit entries, or the diagnosis code was incorrect. After excluding 172 children with tympanostomy tubes or a concomitant diagnosis that affected the antimicrobial choice, a total of 1,240 children were included in the analysis

**Fig. 2:**
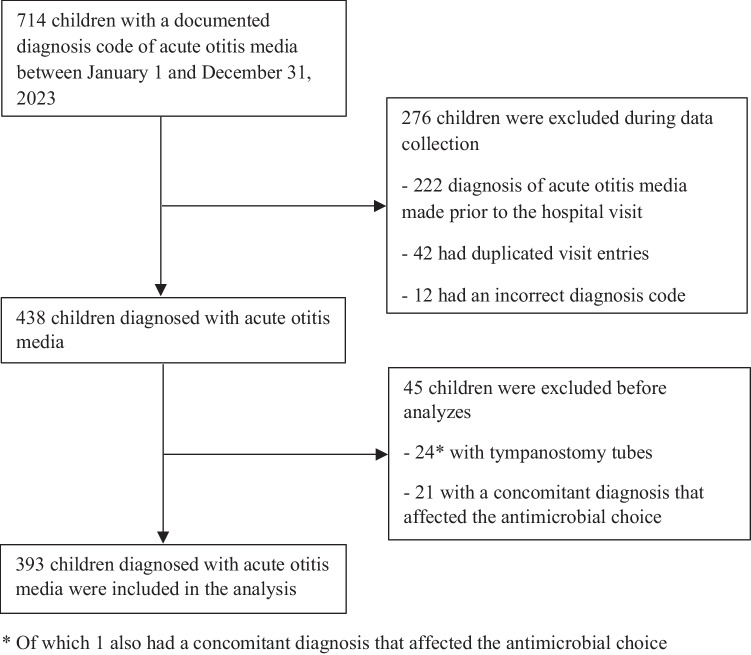
Figure 2 shows the flowchart of the study population in Vaasa. Of the 714 identified children with a diagnosis of acute otitis media in 2023, 276 children were excluded because the diagnosis had been made prior to the hospital visit, they had duplicated visit entries, or the diagnosis code was incorrect. After excluding 45 children with tympanostomy tubes or a concomitant diagnosis that affected the antimicrobial choice, a total of 393 children were included in the analysis

During the study period, a total of 1,595 children in Turku and 714 children in Vaasa with an ICD-10 code for AOM were identified (Figs. 1 and 2). 183 children (11.5%) in Turku and 276 (38.7%) in Vaasa were excluded because the AOM diagnosis had been made prior to the hospital visit, duplicated visit entries, or the diagnosis code was incorrect. A much higher proportion were excluded in Vaasa than in Turku as the registry search in Vaasa included scheduled ENT consultations due to recurrent otitis media without a new diagnosis of AOM. The duplicated visit entries were due to separate diagnoses originating from different specialties in the ED. We also excluded 172 (12.2%) children in Turku and 45 (10.3%) children in Vaasa with tympanostomy tubes or a concomitant diagnosis that affected the antimicrobial choice. Thus, 1,240 children in Turku and 393 children in Vaasa were included in the final analyses.

In Turku, the median age of children was 2.0 years and 55.7% of them were male (Table 1). In Vaasa, the median age was 3.0 years and 54.2% were male. Altogether 711 of 1,240 (57.3%) children in Turku and 210 of 393 (53.4%) children in Vaasa had fever. Most of the children, 993 (80.1%) in Turku and 265 (67.4%) in Vaasa, had had respiratory symptoms. Conjunctivitis was rare, 41 (3.3%) in Turku and 12 (3.1%) in Vaasa. Bulging tympanic membrane was recorded in 670 (54.0%) children in Turku and 193 (49.1%) children in Vaasa. However, tympanic membrane findings were not documented in many of the patient charts (42.1% in Turku and 46.6% in Vaasa). Perforation of the tympanic membrane was documented in 61 (4.9%) children in Turku and 14 (3.6%) in Vaasa. 104 (8.4%) children in Turku and 20 (5.1%) in Vaasa had otorrhea. Antimicrobial allergies were documented in 1.9% of children in Turku and 2.5% in Vaasa.

**Table 1:** Demographics, symptoms and otoscopic findings of the study populations

	Children in Turku (*n* = 1,240)	Children in Vaasa (*n* = 393)
Male sex, *n* (%)	691 (55.7)	213 (54.2)
Age in years, median (IQR)	2.0 (1.0–5.0)	3.0 (1.0–6.0)
Age groups, *n* (%)		
< 1 year	247 (19.9)	32 (8.1)
1–2 years	449 (36.2)	145 (36.9)
3–5 years	286 (23.1)	104 (26.5)
6–9 years	163 (13.1)	69 (17.6)
10–15 years	95 (7.7)	43 (10.9)
Chronic disease, *n* (%)^1^	227 (18.3)	67 (17.0)
Previous acute otitis media, *n* (%)^2^	393 (31.7)	127 (32.3)
Fever (at home or in the emergency department), *n* (%)^3^	711 (57.3)	210 (53.4)
Maximum temperature (ºC), mean (SD)	38.9 (0.7)	38.9 (0.6)
Respiratory symptoms, *n* (%)^3^	993 (80.1)	265 (67.4)
Conjunctivitis, *n* (%)^3^	41 (3.3)	12 (3.1)
Otoscopy, *n* (%)		
Retracted tympanum	4 (0.3)	3 (0.8)
Normal tympanum	44 (3.5)	14 (3.6)
Bulging tympanum	670 (54.0)	193 (49.1)
Not specified or data missing	522 (42.1)	183 (46.6)
Perforated tympanum	61 (4.9)	14 (3.6)
Otorrhea	104 (8.4)	20 (5.1)
Bilateral acute otitis media, *n* (%)^4^	421 (34.0)	98 (24.9)
Antimicrobial allergy, *n* (%)	23 (1.9)	10 (2.5)
Inpatient care, *n* (%)	71 (5.7)	43 (10.9)

^1^Such as atopic dermatitis, allergies, asthma, different neurological disorders and a broad spectrum of other conditions

^2^Data were missing for 723 (58.3%) children in Turku and 247 (62.8%) children in Vaasa

^3^Symptoms reported in the medical record

^4^Data were missing for 29 (2.3%) children in Turku and 6 (1.5%) children in Vaasa

Altogether 80.9% (1,003/1,240) of children in Turku and 80.7% (317/393) in Vaasa were treated with amoxicillin (Table 2). In total, 10.1% (125/1240) in Turku and 8.7% (34/393) in Vaasa received amoxicillin-clavulanate. Other antibiotics were uncommonly prescribed. The antimicrobial treatment was unspecified in a few cases due to incomplete documentation (Turku 4.4%, Vaasa 1.5%).

**Table 2:** Treatment choice for children with acute otitis media

	Children in Turku (*n* = 1240)	Children in Vaasa (*n* = 393)	Absolute difference (%), 95% CI
Watchful waiting, *n* (%)	15 (1.2)	11 (2.8)	−1.6 (−3.0 to −0.2)
Safety-Net Antibiotic Prescription, *n* (%)	56 (4.5)	11 (2.8)	1.7 (−0.5 to 4.0)
Antimicrobial treatment, *n* (%)^1^	1225 (98.8)	382 (97.2)	1.6 (0.2 to 3.0)
Amoxicillin	1003 (80.9)	317 (80.7)	0.2 (−4.2 to 4.7)
Amoxicillin-clavulanate	125 (10.1)	34 (8.7)	1.4 (−1.9 to 4.8)
Phenoxymethylpenicillin	< 5^2^	< 5^2^	N/A
Azithromycin	< 5^2^	< 5^2^	N/A
Sulfamethoxazole-trimethoprim	27 (2.2)	8 (2.0)	0.1 (−1.5 to 1.8)
Cephalexin	9 (0.7)	9 (2.3)	−1.6 (−2.7 to –0.4)
Ceftriaxone^3^	< 5^2^	0 (0)	N/A
Antimicrobial treatment, agent not documented	55 (4.4)	6 (1.5)	2.9 (0.8 to 5.1)
Treatment duration, *n* (%)			
1 day^3^	< 5^2^	0 (0)	N/A
5 days	650 (52.4)	148 (37.7)	14.8 (9.1 to 20.4)
5 to 7 days	22 (1.8)	13 (3.3)	−1.5 (−3.2 to 0.1)
7 days^2^	225 (18.1)	123 (31.3)	−13.2 (−17.8 to −8.5)
10 days^2^	< 5^2^	9 (2.3)	N/A
Duration not documented	337 (27.2)	89 (22.6)	4.5 (−0.5 to 9.5)
Revisit within one week, *n* (%)	63 (5.1)	24 (6.1)	−1.0 (−36 to 1.5)
Revisit, changed treatment, *n* (%)	13 (1.0)	3 (0.8)	0.3 (0.8 to 1.4)

^1^Including children with safety-net antibiotic prescriptions

^2^Data have been anonymized and not displayed in accordance with the Finnish Data Protection Act

^3^Treatment with one dose of ceftriaxone intramuscular because of vomiting

Amoxicillin and amoxicillin-clavulanate are recommended first-line treatments. Sulfamethoxazole-trimethoprim and azithromycin are recommended options in case of penicillin allergy. The recommended antimicrobial treatment duration is 5–7 days

The most frequent length of the antimicrobial treatment was 5 days in both Turku (52.4%) and Vaasa (37.7%), followed by 7 days (Turku 18.1%, Vaasa 31.3%). A 5-day course of treatment was prescribed more often in Turku than in Vaasa (absolute difference 14.8%, 95% CI: 9.1 to 20.4). A small proportion of prescriptions were for 5 to 7 days (Turku 1.8%, Vaasa 3.3%), letting the guardian decide the exact duration depending on the child’s overall recovery. A 10-day course of therapy was uncommon in both hospitals (Turku < 0.4%, Vaasa 2.3%). Watchful waiting was initiated only for 1.2% in Turku and 2.8% in Vaasa. Safety-net antibiotic prescriptions were prescribed to 4.5% of children in Turku and 2.8% in Vaasa. 5.1% in Turku and 6.1% in Vaasa had a return visit within one week after the index AOM encounter date. Treatment failure was rare and documented only in 1.0% of children in Turku and 0.8% in Vaasa.

Most children (302/393, 76.8%) in Vaasa were treated by GPs, 15.3% (60/393) by residents in Pediatrics, 5.6% (22/393) by specialists in Pediatrics and 2.3% (9/393) by ENT physicians. GPs prescribed amoxicillin to 78.8% and amoxicillin-clavulanate to 9.3% of the children with AOM. The corresponding proportions for amoxicillin prescriptions were 95.0% for residents in Pediatrics, 81.8% for specialists in Pediatrics, and 44.4% for ENT physicians, respectively.

## Discussion

This study shows that amoxicillin is clearly the most frequently prescribed antimicrobial agent for the treatment of AOM in the two hospitals studied. In both Turku and Vaasa, amoxicillin was prescribed in more than 80% of the cases. Amoxicillin-clavulanate was the second most prescribed antibiotic for AOM, accounting for 9–10% of cases. Alternative antibiotics were rarely prescribed. A short course of 5–7 days treatment was prescribed to almost all children. The treatment used appears to be effective, since treatment failure was rare.

Amoxicillin was the most prescribed antimicrobial treatment even if amoxicillin-clavulanate is an equally acceptable treatment alternative according to the Finnish guidelines [7]. The American Academy of Pediatrics recommends amoxicillin-clavulanate as a first-line option only in selected cases, such as children with conjunctivitis, prior amoxicillin exposure within the last month, or recurrent or resistant AOM 3. The Finnish guidelines allow choosing between the two treatment agents based on the child’s condition, the drug’s side effect profile, and local antibiotic resistance [7]. Although both amoxicillin and amoxicillin-clavulanate have been compared with placebo [8–15], they have not been directly compared with each other in the treatment of AOM in any randomized controlled trial [16]. Therefore, it remains uncertain which treatment is superior, and both have advantages. Amoxicillin has a narrow spectrum and fewer side effects compared to amoxicillin-clavulanate [17–19]. On the other hand, amoxicillin-clavulanate is effective against beta-lactamase-producing bacteria, such as *Moraxella catarrhalis* and some strains of *Haemophilus influenzae,* which were the most common pathogens detected by PCR from middle ear fluids in Finnish children with AOM (47% and 33%, respectively) [20]. In 2023, 32% of *Haemophilus influenzae* strains isolated from young Finnish children were resistant to amoxicillin [21]. The recommended amoxicillin dose, 40 mg/kg/day, is lower in Finland than in many countries [2–5, 7], since pneumococcal penicillin resistance is relatively rare in Finland [21, 22]. Other Nordic countries recommend penicillin as the first line treatment [23–25], which has even narrower antimicrobial spectrum than amoxicillin.

The most frequently prescribed treatment duration in both hospitals was 5 days. A 5-day course was more often prescribed in Turku than in Vaasa. We assume that a larger proportion of physicians in Turku worked as pediatricians, who tend to use a 5-day antibiotic regimen more commonly than GPs. In Vaasa most of the physicians where GPs. However, a short 5- to 7-day treatment regimen was prescribed in nearly all cases in both Turku and Vaasa, which is in line with the Finnish guidelines. Evidence from a randomized controlled trial has showed that a 10-day antibiotic regimen was more effective than a 5-day course in resolving symptoms and preventing recurrence for children ages 6–23 months with AOM [26], but there are several other studies indicating that a 5-day treatment is also effective [27–31]. The evidence comparing the efficacy of 5-day versus 7-day treatment duration is lacking. The American Academy of Pediatrics recommends short 5- to 7-day durations for children aged 2 years and older with non-severe and uncomplicated AOM [3]. Guideline adherence varies across studies. In the US, only 57–83% of antibiotic prescriptions for AOM involved first-line agents, and most children, also aged 2 years or older, received a 10-day treatment [32–37]. One study reported that European primary care pediatricians prescribed amoxicillin in 89% of AOM cases treated with antibiotics [38]. The Finnish guidelines appear to be well followed in the two hospitals studied, as approximately 90% of the prescriptions were for one of the two recommended treatments, and almost all durations for the recommended 5–7 days.

Most of the children were treated with antibiotics. This is also in line with the Finnish guidelines, which generally recommend antibiotic treatment once the diagnosis of AOM has been confirmed 7. However, a watchful waiting strategy is also an option, with reassessment of the child within 2–3 days, if no clear clinical improvement has been observed 7. Safety-net prescriptions are not mentioned in the Finnish guidelines [7]. In a large-scale retrospective study involving children diagnosed with AOM in private primary care clinics in Finland, antibiotics were prescribed to only 45% of all children with AOM [39]. However, some of these visits were likely follow up appointments for AOM (i.e. not new episodes), because in the past, these were routinely recommended in Finland. In our study, all children presented with a new episode of AOM. Watchful waiting has been demonstrated in several studies to be an effective alternative to immediate antibiotic treatment [10, 15, 40–43] and is recommended as a primary treatment option in most European guidelines [2]. In this respect, the Finnish guidelines differ markedly from those of other Nordic countries, which predominantly recommend watchful waiting for children over 6 months or 1 years of age with uncomplicated AOM [23–25]. It has been estimated that more than 5,000 children need to be treated with antibiotics to prevent one case of acute mastoiditis [44]. No increase in the incidence of acute mastoiditis was observed after Sweden adopted watchful waiting in their national guidelines [45]. Our results raise the question of whether Finnish physicians are sufficiently familiar with the watchful waiting. This is something that we propose should be revised when the Finnish guidelines are updated, since it remains an important strategy to minimize unnecessary antibiotic use. According to a study conducted in 2019, Finnish parents seem to accept watchful waiting as a treatment option for AOM [46].

Turku University Hospital is a tertiary care hospital within the Wellbeing Services County of Southwest Finland, while Vaasa Central Hospital is a secondary care hospital within the Wellbeing Services County of Ostrobothnia. Both hospitals provide specialized healthcare service within the Finnish public healthcare, with their EDs offering both primary and specialized care. The Wellbeing Services County of Southwest Finland has approximately three times the population of the Wellbeing Services County of Ostrobothnia, which is consistent with the proportion of patients in our material. Most children in Vaasa were diagnosed by GPs. Residents in Pediatrics prescribed amoxicillin to nearly all children with AOM and GPs to 79%. In the study from the private primary care clinics in Finland, amoxicillin was also the most frequently prescribed first-line antibiotic for AOM, even if the proportion was markedly lower than in our study, with GPs prescribing it in about half of the cases and specialists in Pediatrics in only 40% [39].

Most of the visits within one week after the index AOM encounter date did not lead to a change in antimicrobial treatment. Treatment failures were rare and less frequent than in a large retrospective cohort study conducted in the US with predominantly 10-day treatments [35], indicating that the initial management was successful in most cases. The reliability of the diagnosis may influence this. Specified tympanic membrane findings were missing in many cases (58–63%), although it was documented that AOM was clinically diagnosed. We assume that most cases were correctly diagnosed, but this cannot be retrospectively confirmed. It is also likely that more children had respiratory symptoms than was documented, as AOM is almost always preceded or accompanied by an upper respiratory tract infection [47].

This study has certain limitations. The data were collected retrospectively. In some cases, the medical documentation was incomplete. AOM diagnosis was based on clinical findings and otoscopy, which are subjective, making it impossible to assess their accuracy. We were not authorized to use the electronic prescription database or Kanta, the Finnish electronic health service containing all healthcare information. Thus, we could not determine whether children had any return visits to other healthcare centers or filled prescriptions for delayed treatment. Overall, we could not confirm whether the children really used the prescribed antimicrobial treatment or for how long.

The main strengths of this study are that all children under the age of 16 years with an AOM diagnosed in Turku University Hospital or Vaasa Central Hospital during 2023 were included in the study, and all information was collected separately for each child by an individual review of the electronic patient charts. In Finland, emergency services are freely accessible for all children, insured or not, ensuring equal healthcare access. Thus, the study population was heterogeneous in terms of both background characteristics and AOM severity, as we included children with otorrhea or perforations. Although our findings are not directly applicable to other countries, they indicate that a short course of amoxicillin may be adequate for treatment of AOM.

## Conclusion

Our results show that most cases of AOM were treated with a 5-day amoxicillin regimen, followed by amoxicillin-clavulanate as the second most frequently prescribed antimicrobial treatment. This indicates that compliance with the Finnish guidelines for AOM is good in the two Finnish hospitals studied. On the other hand, only few children were treated with watchful waiting, which is important to take into account when the Finnish guidelines are updated. Given the forthcoming revision of the Finnish guidelines, it would be important to examine how potential changes in the recommendations are implemented in practice.

## Data Availability

Registry-based data cannot be freely shared with other research groups due to the restrictions under the Finnish Data Protection Act.

## Abbreviations

AOM: Acute otitis media
CI: Confidence interval
ED: Emergency department
GP: General practitioners
ENT: Ear, nose and throat
The US: The United States
